# Diagnostic Accuracy of Automated Diabetic Retinopathy Image Assessment Software: IDx-DR and RetCAD

**DOI:** 10.1007/s40123-024-01049-z

**Published:** 2024-11-06

**Authors:** Andrzej Grzybowski, Piotr Brona, Tomasz Krzywicki, Paisan Ruamviboonsuk

**Affiliations:** 1 Institute for Research in Ophthalmology, Foundation for Ophthalmology Development, Poznan, Poland; 2 Department of Ophthalmology, Poznan City Hospital, Szwajcarska 3, 60-285 Poznan, Poland; 3 Department of Mathematical Methods of Informatics, University of Warmia and Mazury, Olsztyn, Poland; 4 Department of Ophthalmology, Rajavithi Hospital, College of Medicine, Rangsit University, Bangkok, Thailand

**Keywords:** Diabetic retinopathy, Artificial intelligence, Image, Diagnostic, Screening

## Abstract

**Introduction:**

Automated diabetic retinopathy (DR) screening using artificial intelligence has the potential to improve access to eye care by enabling large-scale screening. However, little is known about differences in real-world performance between available algorithms. This study compares the diagnostic accuracy of two AI screening platforms, IDx-DR and RetCAD, for detecting referable diabetic retinopathy (RDR).

**Methods:**

Retinal images from 758 patients with diabetes were collected during screening from various clinics in Poland. Each patient was graded by three graders with 320 patients graded by Polish and 438 patients graded by Indian graders, with the majority decision serving as the reference standard. The images were evaluated independently by the IDx-DR and RetCAD algorithms. Sensitivity, specificity, positive and negative predictive values, and agreement between algorithms and human graders were calculated and statistically compared.

**Results:**

IDx-DR demonstrated higher sensitivity of 99.3% but lower specificity of 68.9% for RDR detection compared to RetCAD which had 89.4% sensitivity and 94.8% specificity. The positive predictive value was higher for RetCAD (96.4% vs 48.1% for IDx-DR) while the negative predictive value was higher for IDx-DR (99.5% vs 83.1% for RetCAD). Both algorithms achieved high sensitivity (> 95%) for sight-threatening diabetic retinopathy detection.

**Conclusion:**

In this direct comparison using the same patient cohort, the two algorithms showed differences in their operating parameters for RDR screening. IDx-DR prioritized avoiding false negatives over false positives while RetCAD maintained a more balanced trade-off. These results highlight the variable performance of current artificial intelligence screening solutions and suggest the importance of considering algorithm performance metrics when deploying automated diabetic retinopathy screening programs, based on available healthcare resources.

## Key Summary Points

**Table:** 

In this direct comparison using the same patient cohort, the two algorithms showed differences in their operating parameters for RDR screening.
IDx-DR prioritized avoiding false negatives over false positives while RetCAD maintained a more balanced trade-off.
These results highlight the variable performance of current artificial intelligence screening solutions and suggest the importance of considering algorithm performance metrics when deploying automated diabetic retinopathy screening programs, based on available healthcare resources.

## Introduction

Diabetes is a worldwide epidemic and one of the world’s fastest growing diseases. Despite widespread public health efforts worldwide, age-standardized mortality rates from diabetes increased by 3% during the last two decades [1]. The number of complications from diabetes, including ophthalmological complications, is similarly expected to climb along with the increased prevalence of diabetes worldwide [2]. Diabetic retinopathy is one of the leading causes of blindness in working age adults [3]. This emphasizes the need for efficient and cost-effective diabetic retinopathy (DR) screening services. Large-scale screening of DR, based on traditional human grading, has so far been out of reach of most regions in the world [4]. This is influenced by the prohibitive financial and human resources required for the development and upkeep of traditional comprehensive DR screening systems [5].

One of the proposed solutions to this issue is to employ an automated, AI-based screening algorithm in the screening process. Automated diabetic retinopathy image assessment systems (ARIAs) are software packages or algorithms capable of grading fundus pictures replacing or assisting human graders, with the hope of enabling accurate, efficient, cost-effective, and scalable DR screening [6]. AI-based DR screening has been shown to increase screening access and health equity [7].

Several ARIAs are now available, with many more being developed throughout the world [8]. While there are a number of research studies examining the performance of a single ARIA [1], studies comparing multiple ARIAs are still uncommon because of the difficulty of direct comparison [1, 9]. Previous research has shown that the performance of even cutting-edge algorithms can vary significantly [5, 10]. Direct comparison of accuracy metrics of different ARIAs from the, relatively plentiful, single-ARIA studies is misleading because of a number of factors including differences in cutoffs for referrable DR and grading protocols, equipment used to take fundus images, differences in accounting for low-quality images or encounters, presenting and comparing results on different levels (per picture or per eye or per patient).

Only three DR screening algorithms received Federal Drug and Food Administration (FDA) approval in the USA, with IDx-DR being the first algorithm to be approved, following a pre-registered, independently run trial [11]. In contrast to the US market, the number of market-approved algorithms worldwide is growing at a fast rate, with over 20 such solutions registered in the European Union (EU) alone and more being developed and brought onto the market each year. At the same time there is a continued scarcity of research and, more importantly, relative lack of independent analysis of the accuracy and performance of those algorithms [12]. This hinders the potential verification, introduction, and improvement of those solutions. When trying to introduce those ARIAs into real-life scenarios, governing bodies and decision-makers have little to go on when choosing a particular solution [13, 14]. Th objective of the current study was to directly compare and contrast two ARIAs on the same dataset. On the basis of the resources and access to algorithms made available to us, we set out to directly evaluate the performance of an established, FDA-approved ARIA—IDx-DR—with another contemporary solution—RetCAD.

## Methods

### Study Design

In this retrospective comparative study, the performance of two different ARIAs in screening for DR was compared to that of human graders (reference standard). The screening for DR was conducted and retinal images were obtained from diabetic clinics in Poznan, Poland between March 2020 and April 2021. All the extracted images were anonymized and deidentified, no change in clinical pathway was anticipated, and the study followed the principles of the declaration of Helsinki. The study was approved by the Institutional Review Board at Foundation for Ophthalmology Development, approval no. nr 2/2024.

The primary objective of the study was to assess the sensitivity and specificity of ARIAs in detecting referable diabetic retinopathy (RDR). The secondary objectives were to assess the positive and negative predictive values of ARIAs to detect RDR and to assess the sensitivity and specificity of ARIAs for detecting sight-threatening diabetic retinopathy (STDR). Additionally, kappa agreement was measured between the two ARIAs and the reference graders. Statistical differences between classifiers were checked using the McNemar test [15].

### Sample Size

Using an alpha error of 0.05, a precision rate of 10% (two sided), an estimated sensitivity of 85%, and an estimated incidence of RDR (ICDR; moderate non-proliferative diabetic retinopathy (NPDR) and/or presence of macular edema) of 7%, we calculated the sample size to be 700 participants. Given these assumptions and expecting that at least 10% of subjects may be qualified as insufficient in quality by the AI systems, a sample size of 781 subjects was chosen.

### Inclusion and Exclusion Criteria

The retinal images of subjects with established diabetes mellitus that were captured at the time of DR screening were eligible for inclusion in the study. Images of subjects that did not have at least one disc and one macula-centered image of sufficient quality (as deemed by the IDx-DR quality check process) were excluded for the study. Additionally, subjects who received treatment for DR (lasers or intraocular injections) were excluded. In total 107 patients were excluded from the study on the basis of insufficient quality.

### Retinal Image Acquisition

The screening process involved non-mydriatic fundus images captured using a Topcon camera Nw-400 by trained operators. For each patient, at least four images (45° field of view each) which included two sets of one image centered on the optic disc and one centered on the macula were captured. Additional images were taken to ensure sufficient quality, using the IDx-DR built-in image quality feedback system. Retinal images were obtained from 779 consecutive patients with established diabetes mellitus with sufficient quality deemed by IDx-DR. After patient exclusion due to insufficient quality by the RetCAD AI, 21 patients additional patients were excluded. A total of 3427 images of sufficient quality from 758 patients were used for the study.

Unfortunately, as a result of the design and protocol of the screening program from which the images were taken for this analysis, we only had access to images of patients that successfully completed screening with IDx-DR. In effect all images analyzed in this study were taken from encounters deemed as sufficient by the IDx-DR quality check. Because of this we could not fairly compare the ability of both systems to deal with and designate low-quality encounters and chose to further analyze only images accepted by both systems.

### Reference Standard Grading

The patients with images with sufficient quality were split into two sub-datasets. 320 patients were graded by three Polish retinal specialist graders and 438 by three certified graders in India. All three polish graders were consultant ophthalmologists working in the field of retinal diseases (Poland has no formal subspeciality training for ophthalmologists).The three Indian graders were two retina specialists and one certified optometrist grader. We determined the agreement of grades between group of graders using Fleiss’ kappa [16] coefficient (Table 1) along with the agreement between graders and the reference standard using Cohen’s kappa [17] coefficient. All the graders were masked to the output of the AI and to each other’s grading. Images were graded for severity of diabetic retinopathy based on International Clinical Diabetic Retinopathy (ICDR) severity classification as no DR, mild Non-proliferative Diabetic Retinopathy (NPDR), moderate NPDR,severe NPDR and Proliferative Diabetic retinopathy (PDR). Macular edema was determined by the presence of surrogate markers like hard exudates. If hard exudates were found within 1 DD of the fovea, macular edema was determined as significant and labelled as clinically significant diabetic macular edema (CSDME) present. Image grading was done on a pereye basis. The final diagnosis for each patient was determined by the stage of DR of the more affected eye. Consensus image grading was regarded as the final reference standard based on Polish and Indian graders for the comparison of both AI systems. All the analysis was performed at the patient level.

**Table 1:** Values of Fleiss kappa coefficients depicting agreement of graders

	Fleiss’ kappa
Polish	0.44
Indian	0.54

### Definitions

Referable diabetic retinopathy was defined as moderate NPDR and more severe disease (moderate NPDR, severe NPDR, PDR) and/or the presence of CSDME. STDR was defined as severe NPDR or PDR and/or the presence of CSDME.

### Artificial Intelligence Analysis Using Automated Grading Systems

We used two different ARIAs, i.e., RetCAD v2.1.1 (Thirona Retina BV, Nijmegen, the Netherlands) and IDx-DR (vx.y.z) (Digital Diagnostics, Iowa, USA). The retinal images were run on both the ARIAs to screen for DR. IDx-DR results were recorded during live screening and all images captured for the patient were analyzed on a per patient basis. Two images per eye that passed the AI quality check were submitted to the AI for DR analysis. For RetCAD analysis, anonymized images for each patient were securely transferred to a cloud platform and were processed automatically. On-premise analysis using RetCAD would be possible but as a result of the study setup, a cloud version of the RetCAD AI was used.

**Fig. 1:**
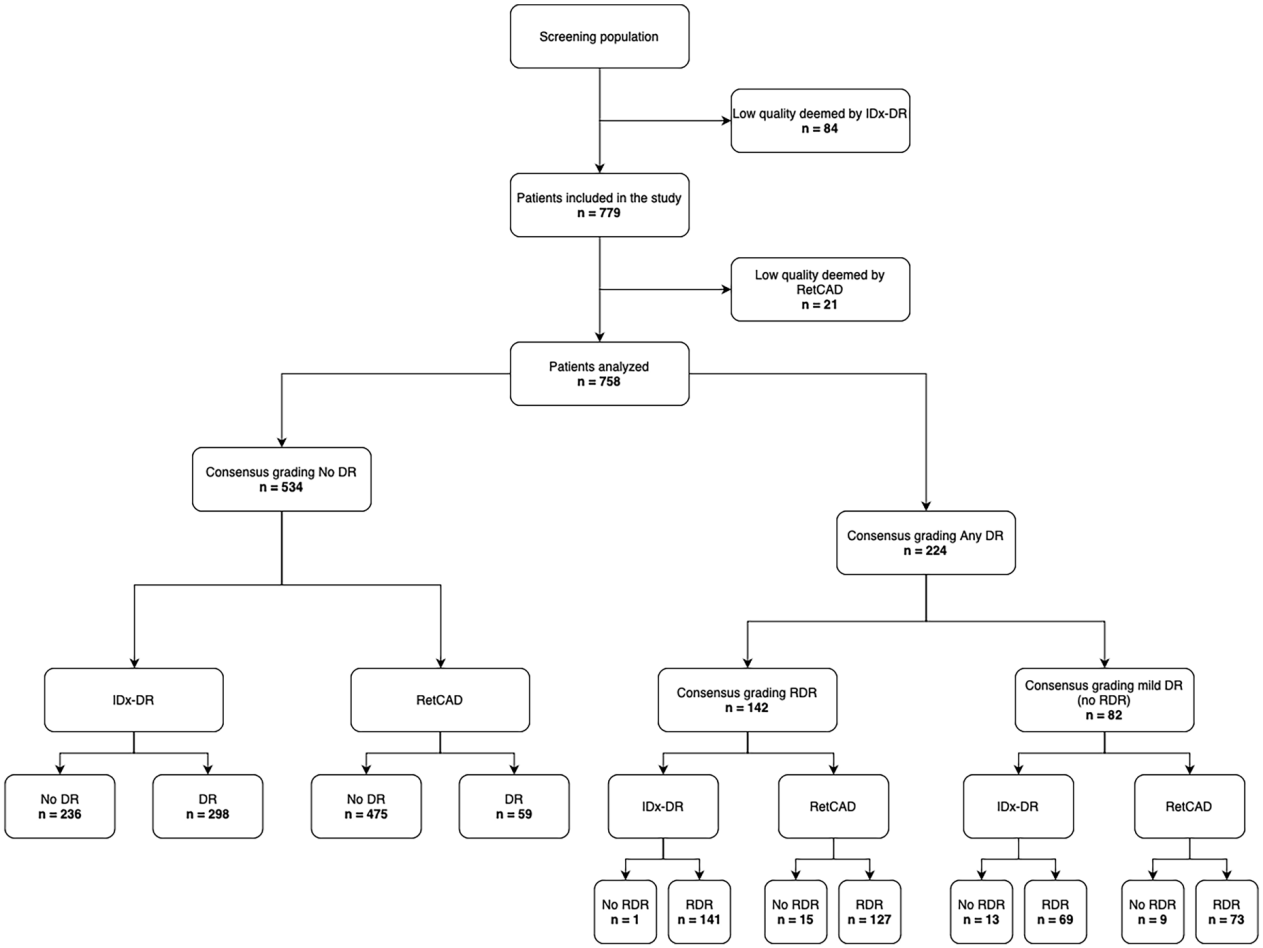
Classification diagram showing the patient breakdown for the RDR analysis. *DR* diabetic retinopathy, *RDR* referrable diabetic retinopathy

Both the AI systems are based on convolutional neural networks (CNN). The output of the model was a real continuous number ranging from 0 to 4.0 corresponding to the severity of DR. A report was generated on a per patient basis. The IDx-DR system outputs the stage of DR and generates a per patient report. Figure 1 depicts the outputs generated from the IDx-DR AI system. Table 2 shows the mapping of the RetCAD software to ICDR stages.

**Table 2:** Mapping of the RetCAD software to ICDR stages

RetCAD AI score	ICDR level
0.00–0.49	No DR
0.50–1.49	Mild DR
1.50–2.49	Moderate DR
2.50–3.49	Severe DR
3.50–4.00	Proliferative DR

IDx-DR was used on a commercial basis, as it was used in the screening program from which the images and IDx-DR results are sourced. RetCAD analysis was made available to us free of charge for the purpose of this study.

### Statistical Analysis

All data was stored in CSV files and was analyzed using Python programming languages along with Numpy, Pandas, Scikit learn, and Scipy libraries. The diagnoses of the AI using RetCAD and IDx-DR AI systems were tabulated against the consensus image diagnosis (reference standard) by constructing 2 × 2 confusion matrices. The sensitivity, specificity, positive predictive value (PPV), and negative predictive value (NPV) with 95% CIs were calculated. The agreement between system and gold standard diagnoses was also measured using the kappa coefficient [18]. For comparing the multistage diagnoses of the RetCAD and IDx-DR systems, the DR diagnoses were transformed from the five-stage scale assigned by the RetCAD system and by the gold standard to a four-stage scale because of the scale of the IDx-DR system diagnoses. This was done by joining the two top severity scores from RetCAD, severe DR and proliferative DR, into one STDR category, therefore matching diagnostic output of IDx-DR. An unweighted kappa coefficient was used to measure the concordance of diagnoses binarized at the any DR and RDR stages, and a quadratic weighted kappa coefficient was used to measure the concordance at the four-stage scale. We used the McNemar test with an alpha of 0.05 to assess the statistical significance of differences between classifiers. We performed the tests for scores on a four-class scale and on a binary scale showing the occurrence or lack of any DR, RDR, and STDR.

## Results

From the initial study populations, 84 patients were excluded because of insufficient image quality as determined by IDx, 21 patients were excluded subsequently because of insufficient image quality as determined by RetCAD AI, and an additional 2 patients were excluded as a result of insufficient image quality as determined by the reference graders. This resulted in a total study population of 758 patients. Figure 1 presents the classification diagram of retinal image selection in the study.

Grades from the Polish and Indian graders were converted to No DR, any DR, RDR and STDR before computing consensus. Based on consensus grading, there was no evidence of DR in 534 patients (70%). Any DR was seen in 224 (30%), of which 82 were mild DR (11%), and 142 (19%) were RDR. Of the 142 RDR cases, 36 (5%) were STDR. The inter-rater agreement (Cohen's kappa) for Poland graders was 0.540 (Ophthalmologist 1), 0.764 (Ophthalmologist 2) and 0.502 (Ophthalmologist 3). For the Indian graders, kappa was 0.710 (Ophthalmologist 1), 0.783 (Ophthalmologist 2) and 0.741 (Ophthalmologist 3). These grader variability metrics are quite close to our previous study looking into grader variability [18, 19].

The IDx-DR AI system detected the presence of DR in 508 patients (69%); 332 of them were also categorized as having RDR (44%) and 161 as STDR (21%). The IDx-DR system could detect RDR in 141 patients with a sensitivity of 99.30% (95% CI 96.14%, 99.98%), at a cost of low specificity at 68.99% (95% CI 65.18%, 72.63%). The RetCAD system detected the presence of DR in 214 patients (28%). The RetCAD system was able to detect 127 patients with referable DR (RDR) with a sensitivity of 89.44% (95% CI 83.18%, 93.97%) at a very high specificity of 94.81% (92.75%, 96.42%). The diagnostic ability of both the AI systems including sensitivity, specificity, and positive and negative predictive values is tabulated in Tables 3, 4, 5, 6, and 7.

**Table 3:** Number of patients by DR grade

Based on consensus image grading	Total number of patients (*n*)	Absence of any form of DR	Presence of any type of DR	Presence of referable DR	Presence of sight-threatening DR
Sample (*n*)	758	534	224	142	36

*DR* diabetic retinopathy

**Table 4:** Confusion matrix of the RetCAD system in DR diagnosis

	For any DR		For referable DR		For sight-threatening DR
	DR positive (reference standard)	DR negative (reference standard)	RDR positive (reference standard)	RDR negative (reference standard)	STDR positive (reference standard)
RetCAD positive	214	59	127	32	34
RetCAD negative	10	475	15	584	2

*DR* diabetic retinopathy, *RDR* referrable diabetic retinopathy, *STDR* sight-threatening diabetic retinopathy

**Table 5:** Confusion matrix of the IDx-DR system in DR diagnosis

	For any DR		For referable DR		For sight-threatening DR
	DR positive (reference standard)	DR negative (reference standard)	RDR positive (reference standard)	RDR negative (reference standard)	STDR positive (reference standard)
IDx-DR positive	222	298	141	191	35
IDx-DR negative	2	236	1	425	1

*DR* diabetic retinopathy, *RDR* referrable diabetic retinopathy, *STDR* sight-threatening diabetic retinopathy

**Table 6:** Statistical comparison of RetCAD and IDx-DR

Variables	For any DR		For referable DR		For sight-threatening DR	
	RetCAD	IDx-DR	RetCAD	IDx-DR	RetCAD	IDx-DR
Sensitivity	95.54 (91.94, 97.84)	99.11 (96.81, 99.89)	89.44 (83.18, 93.97)	99.30 (96.14, 99.98)	97.22 (85.47, 99.93)	94.44 (81.34, 99.32)
Specificity	88.95 (85.98, 91.48)	44.19 (39.93, 48.52)	94.81 (92.75, 96.42)	68.99 (65.18, 72.63)	82.55	95.01
PPV	78.39 (74.00, 82.81)	42.69 (40.83, 44.57)	79.87 (73.82, 84.82)	42.47 (39.60, 45.39)	21.74 (19.02, 24.73)	48.57 (40.49, 56.73)
NPV	97.94 (96.28, 98.87)	99.16 (96.73, 99.79)	97.50 (96.02, 98.43)	99.77 (98.37, 99.97)	99.83	99.71

Values in % (95% CI)

*PPV* positive predictive value, *NPV* negative predictive value, *CI* confidence interval, *DR* diabetic retinopathy

**Table 7:** Performance metrics for detecting referrable diabetic retinopathy separated by the two grader groups

Variables	Indian graders		Polish graders	
	RetCAD	IDxDR	RetCAD	IDx
Sensitivity	87.18 (79.74, 92.64)	99.15 (95.33, 99.98)	100.00 (86.28, 100.00)	100.00 (86.28, 100.00)
Specificity	97.56 (95.25, 98.94)	68.60 (63.27, 73.58)	91.67 (87.85, 94.59)	69.44 (63.77, 74.71)
PPV	92.73 (86.50, 96.21)	52.97 (48.95, 56.95)	51.02 (51.53, 60.44)	22.12 (19.27, 25.27)
NPV	95.52 (93.00, 97.16)	99.56 (96.96, 99.94)	100 (98.61, 100.00)	100.00 (98.17, 100.00)

Values in % (95% CI)

*PPV* positive predictive value, *NPV* negative predictive value, *CI* confidence interval, *DR* diabetic retinopathy

## Discussion

In this study, our primary objective was to evaluate the sensitivity and specificity of two selected ARIAs, IDxDR and RetCAD, in detecting RDR, while our secondary objectives encompassed assessing the positive and negative predictive values of the ARIAs for RDR detection and evaluating its sensitivity and specificity in identifying STDR.

For our primary goal and the secondary goal concerning RDR detection, we found that IDx-DR exhibits a higher sensitivity at the cost of substantially lower specificity score, 95.9% and 68.5%, respectively. In contrast, RetCAD demonstrates a more balanced approach with high sensitivity and specificity, 88.0% and 94.7%. The trade-off between PPV and NPV is more spread: for IDX the NPV is 99.5% and the PPV is 48.1%; for RetCAD the NPV is 83.1% and the PPV is 96.4%. In a previous study RetCAD achieved similar results with 80.0% sensitivity and 90.1% specificity; however, that study was done on the general population visiting an ophthalmology clinic, and not only on patients with diabetes; therefore, only 31 out of 1245 eyes were deemed DR positive by expert graders [20]. IDx-DR has been validated in a large prospective, independently run trial achieving 87.2% sensitivity and 90.7% specificity, overall a more balanced result compared to the sensitivity-favored accuracy found in this study [11].

When separating the study results according to the two grading groups, Polish and Indian, the overall trends remain the same (Table 5). Both systems had high sensitivity in each grading group; however, RetCAD had slightly lower sensitivity in the Indian grader arm of the study (87.2% RetCAD vs 99.2% IDx-DR), which decreased the overall sensitivity. Specificity metrics for both ARIAs were very similar between the two grading groups.

There have been very limited studies comparing different ARIAs on the same dataset. We previously compared the performance of IDx-DR and RetinaLyze on a limited dataset of 170 patients [5]. That study was done on a similar demographic to this study—patients presenting to a single Polish diabetic clinic—and the sample was non-representative. Sixty DR-positive and 110 DR-negative patients were chosen, with only a single clinician reading all the images to set the ground truth. The study found that the agreement rate with the grader for DR-positive and DR-negative cases was 93.3% and 95.5% for IDx-DR, and between 74.1% and 89.7% or 71.8% and 93.6% depending on the strategy chosen for RetinaLyze. More recently Grzybowski and colleagues performed a head-to-head comparison of IDxDR and MediosAI—another ARIA available on the market in Europe [21]. That study was based on 807 Polish patients and based on very similar concepts to the current paper—images taken from an established local DR screening in Poland, graded by three Polish or Indian graders. The results for detecting RDR for IDx-DR were comparable to this study with 99% sensitivity and 68% specificity (99% and 69%, respectively, in this analysis). MediosAI achieved 95% sensitivity and 80% specificity on the same dataset, presenting a detection pattern more shifted towards PPV, similar to RetCAD.

The largest head-to-head comparison of ARIAs to date was published by Lee and colleagues in 2021 [10]. They reached out to 23 companies for the evaluation of their model’s performance, with only five agreeing to participate. Each algorithm was anonymized, and the results were blinded to all researchers and published without naming specific algorithms. The dataset comprised fundus photographs from two Veterans Affairs (VA) hospitals located in the USA. The algorithms’ performance was compared with that of the VA graders. The authors observed a significant variability in model performance across the dataset, with sensitivities ranging from 50.98% to 85.90% and specificities from 60.42% to 83.69% [10]. The models’ performance varied between the two different cohorts, with most algorithms showing lower sensitivity and specificity in a population that was less racially and ethnically diverse and used routine mydriatic screening. As the algorithms’ performance appears to vary significantly, real-life, target population evaluation is crucial [22].

Specific details of real-life implementation like the choice of a particular fundus camera may influence the final accuracy of screening [23]. ARIA accuracy for detecting DME was shown to vary with factors such as patient age or diabetes duration, further emphasizing the need for validating ARIAs on the target population [22, 24].

The results of this study have implications for the workflow of human graders in DR eye screening if these systems were used in a triage tool setting to identify patients who should be seen by a specialist. Firstly, the workload for the second screening stage personnel would have been different. IDX would have reduced the workload from the initial 758 patients to 332, i.e., a reduction by 56.2%, with more than half of the remaining patients not having referrable DR. RetCAD would have led to 142 patients, i.e., a reduction by 81.2%, at the cost of 14 more missed patients.

Secondly, the impact on the healthcare system would be different. With IDX, only 42.7% of the referred patients would genuinely have RDR (PPV), as opposed to 79.9% with RetCAD. With increasing rates of false positives, the second-line workforce is increasingly occupied with patients without signs of RDR, rather than deciding on treatment modalities for patients with RDR.

The differences between the operating parameters and accuracy metrics of the two ARIAs demonstrate a certain fundamental balancing act that is required when designing and optimizing an ARIA. When developing and calibrating such systems, one must carefully balance the cutoff parameters between a positive and negative result. On one side it is crucial to avoid false-negative results, which could have deleterious implications for the patient, on the other side a large number of false positives places a larger burden on healthcare resources, such as secondary graders and ophthalmologists verifying those results and can undermine the public’s and other medical professionals’ trust in the reliability of screening [25].

With the enhanced screening capabilities enabled through AI-based screening, a higher throughput of patients can be expected. Considering the high healthcare system costs associated with false positives, including the need for additional workload by human graders, a balanced trade-off between false-positive referrals and false negatives is generally expected. RetCAD’s lower false-positive rate (for RDR) of 5.2% is balanced by the cost of a higher false-negative rate of 10.6%. The respective values for IDX, of 31.0% and 0.7%, signify a threefold increase in false-positive results, while avoiding a significant number of missed diagnoses. Which approach is more desirable is a complex question without an easy answer. False positives generate additional cost and workload as described above; however, false negatives also generate an additional economic burden related to increased morbidity, days off work, or outright work disability [26]. Moreover, for a new and highly debated piece of technology to be introduced into a healthcare system, most would prefer minimizing potential false negatives because of the associated loss of trust and poor public image.

Regarding the secondary goals concerning the sensitivity and specificity of ARIAs in detecting STDR, we observed that both IDX and RetCAD exhibit very high sensitivity scores, with a marginal difference of four percentage points, and the scores falling within the 95% confidence intervals of the respective other system. That is important to the accuracy metrics for those calculations are hindered by overall small sample size and low prevalence of STDR, resulting in only 36 patients with STDR according to the ground truth.

The Cohen’s kappa representing the intergrader reliability of AI system diagnoses with the reference standard shows that RetCAD has a strong agreement with the reference standard whereas IDX only achieves a fair and moderate one [27].

This study has limitations, which should be addressed in future investigations. The first issue stems from the data acquisition process. As quality assessment of IDx-DR was performed during patient enrollment, this introduces bias in the patients that were included in this study. This prevented any meaningful comparison of the quality assessment of each ARIA, and can have a significant influence on the real-life implementation of screening programs facilitated by AI. Additionally, it hampered the practical implications of the performance comparison of the ARIAs when used in clinical practice. In clinical practice, when a patient with insufficient quality is observed, action should be taken and one should refer that patient for manual intervention. If the AI deemed a patient examination of insufficient quality, one could thus consider treating this patient as a referable patient. This would have an impact on the specificity of both AI systems as more patients will be falsely referred (assuming most of those patients are of healthy condition).

The introduction of two separate teams of graders and subsequent splitting of the datasets does raise concern regarding the ground truth setting. It would be ideal to have each grader evaluate each patient, enhancing the reliability of the results. Splitting the dataset between Polish and Indian graders may decrease the consistency of ground truth setting. Moreover an additional adjudication process, as was sometimes successfully implemented in other studies, could be beneficial, particularly so if the number of graders for each patient was to be increased [18]. The split between Indian and Polish graders was done because of financial constraints. As this study is not funded or renumerated in any way, we relied on grading that was provided to us free of charge, and had to rely on two teams to reach the set sample size. Of course, setting a higher sample size would be largely beneficial; however, that was not feasible for this study because of the aforementioned constraints.

As the available output of the IDx system was a four-class classification, no receiver operating characteristics (ROC) analysis could be done. For RetCAD, the output was on a continuous scale from 0.0 to 4.00, which would allow for ROC analysis. Inclusion for a ROC analysis would allow for a direct comparison of the two AI systems at given specificity or sensitivity values as these can be chosen by changing the operating cutoffs of the system for RDR detection. Additionally, the area under the ROC would give a single metric indicating the overall performance of the system, which is often used for assessment of AI systems.

## Conclusion

Two AI systems have been analyzed and compared in a head-to-head fashion on the same patients. Both AI systems achieved high sensitivity; the RetCAD AI system maintained a significantly higher specificity, at a cost of slightly lower sensitivity compared to the IDxDR system. Differences in operating parameters should be taken into account when designing and deploying screening programs. Ideally, available ARIAs should be tested on the target population prior to deployment, with the most suitable solution for that specific population being chosen. In a second step resources should be prepared according to the specific deployment circumstances taking into account the model’s operating parameters and its specific requirements and intricacies. For models with a higher false-positive rate, like IDx-DR in this study, more resources should be allocated for verification of screening-positive patients. Higher numbers of false-negative diagnoses on the other hand necessitate additional safety netting steps, like additional grading of images by experts, for patients with poor systemic control or earlier rescreening time for such patients.

## Data Availability

The datasets generated during and/or analyzed during the current study are available from the corresponding author on reasonable request.
